# An Account of Diseases in an Eastern District of London, from Sept. 20 to Oct. 20, 1810

**Published:** 1810-11

**Authors:** 


### *An Account of Diseases in an Eastern District of London, from Sept. 20 to Oct. 20, 1810.*

| ACUTE DISEASES. | |
|---|---|
| Cholera | 5 |
| Variola | 8 |
| Rubeola | 7 |
| Pneumonia | 2 |
| Rheumatismus Acutus | 3 |

| CHRONIC DISEASES. | |
|---|---|
| Catarrhus | 5 |
| Tussis | 10 |
| Dyspnœa | 7 |
| Tussis cum Dyspnœa | 8 |
| Phthisis Pulmonalis | 3 |
| Hæmoptysis | 4 |
| Hydrothorax | 2 |
| Anasarca | 3 |
| Palpitatio Cordis | 1 |
| Ascites | 3 |
| Hepatitis Chronica | 2 |
| Diarrhæa | 14 |
| Enterodynia | 5 |
| Dyspepsia | 7 |
| Gastrodynia | 6 |
| Amenorrhœa | 3 |
| Chlorosis | 2 |
| Menorrhagia | 2 |
| Rheumatismus Chronicus | 6 |

| PUERPERAL DISEASES. | |
|---|---|
| Menorrhagia Lochialis | 3 |
| Stranguria | 2 |
| Ephemera | 3 |
| Dolor post Partum | 5 |

| INFANTILE DISEASES. | |
|---|---|
| Aphthæ | 2 |
| Pertussis | 4 |
| Tabes Mesenterica | 2 |
| Scrophula | 1 |
| Tinea | 2 |

The uncommonly fine weather which we have lately enjoyed, seems to have carried us back to the season of summer, rather than to have conveyed us through the autumnal quarter towards winter. From the state of atmosphere and the temperature of the air, which generally prevails at this season of the year, it is natural to expect the appearance of those diseases which are connected with it, and which constitute the general epidemic of the autumnal period. But as the weather, which is usually expected at this time of the year, has been protracted, so the appearance of diseases which usually accompany it has also been protracted, or at least they have been attended by a train of symptoms unusually mild. Complaints of the bowels have indeed been frequent. Diarrhæas have been numerous, but they have seldom proceeded beyond the point at which they are generally found to have some salutary influence upon the constitution; and when, from an impatience to get rid of them, or an apprehension that the patient would be too much weakened by the evacuation, astringents have been had recourse to, they have generally been succeeded by pains in the stomach, oppression about the præcordia, want of appetite, or indigestion. Cholera has occurred in a few instances, but this this has, for the most part, appeared in so mild a form as to require very little more than a plentiful use of warm diluting liquids. In some cases, however, an occasional anodyne has been necessary to quiet the excessive action of the biliary system, or to secure to the patient the advantage of a few hours sleep after the anxieties and fatigues of the day.

